# Purification and characterization of the produced hyaluronidase by *Brucella Intermedia* MEFS for antioxidant and anticancer applications

**DOI:** 10.1186/s12934-024-02469-z

**Published:** 2024-07-18

**Authors:** Mai A. Ebraheem, Esmail M. El-Fakharany, Sherif Moussa Husseiny, Fafy A. Mohammed

**Affiliations:** 1 Botany Department, Faculty of Women for Arts, Science and Education, Ain Shams University, Cairo, Egypt; 2 Protein Research Department, Genetic Engineering and Biotechnology Research Institute GEPRI, City of Scientific Research and Technological Applications (SRTA-City), New Borg El-Arab 21934, Alexandria, Egypt; 3 Pharmaceutical and Fermentation Industries Development Centre (PFIDC), City of Scientific Research and Technological Applications (SRTA-City), New Borg El-Arab 21934, Alexandria, Egypt; 4 Pharos University in Alexandria, Canal El Mahmoudia Street, Beside Green Plaza Complex 21648, Alexandria, Egypt

**Keywords:** Microbial hyaluronidase, Production and purification, *Brucella Intermedia*, Antioxidant and antitumor

## Abstract

Hyaluronidase (hyase) is an endoglycosidase enzyme that degrades hyaluronic acid (HA) and is mostly known to be found in the extracellular matrix of connective tissues. In the current study, eleven bacteria isolates and one actinomycete were isolated from a roaster comb and screened for hyase production. Seven isolates were positive for hyase, and the most potent isolate was selected based on the diameter of the transparent zone. Based on the morphological, physiological, and 16 S rRNA characteristics, the most potent isolate was identified as *Brucella intermedia* MEFS with accession number OR794010. The environmental conditions supporting the maximum production of hyase were optimized to be incubation at 30 ºC for 48 h and pH 7, which caused a 1.17-fold increase in hyase production with an activity of 84 U/mL. Hyase was purified using a standard protocol, including precipitation with ammonium sulphate, DEAE as ion exchange chromatography, and size exclusion chromatography using Sephacryle S100, with a specific activity of 9.3-fold compared with the crude enzyme. The results revealed that the molecular weight of hyase was 65 KDa, and the optimum conditions for hyase activity were at pH 7.0 and 37 °C for 30 min. The purified hyase showed potent anticancer activities against colon, lung, skin, and breast cancer cell lines with low toxicity against normal somatic cells. The cell viability of hyase-treated cancer cells was found to be in a dose dependent manner. Hyase also controlled the growth factor-induced cell cycle progression of breast cancer cells and caused relative changes in angiogenesis-related genes as well as suppressed many pro-inflammatory proteins in MDA cells compared with 5-fluorouracil, indicating the significant role of hyase as an anticancer agent. In addition, hyase recorded the highest DPPH scavenging activity of 65.49% and total antioxidant activity of 71.84% at a concentration of 200 µg/mL.

## Introduction

Cancer diseases are the second most prevalent cause of mortality worldwide after cardiovascular diseases. According to the recent reports, the number of fatalities caused by cancer will increase to reach 13.1 million until 2030 [1]. Thyroid, lung, cervical, colorectal and breast cancers are the most prevalent malignancies in women, conversely, the most prevalent malignancies in men are prostate, liver, stomach, lung, and colon [2, 3]. Breast cancer is one of the most prevalent cancers in women and the second most common cancer type overall after lung cancer [4]. Generally, the main goal in breast cancer therapy is to remove the tumor surgically and stop metastasis and recurrence by using neoadjuvant and cytotoxic drugs as well as radiotherapy. Although chemotherapy is commonly used to kill tumor cells or at least prevent its reproduction, but it has a severe side effects and cytotoxicity to normal cells. Targeted therapies were used as lower side effects alternate of traditional chemotherapeutic agents because of their capacity to target most cancer cells specifically while shielding healthy cells. Over the past 20 years, the FDA has authorized a significant rise in targeted cancer therapies but most of them recorded adverse effects [5]. Tamoxifen, very successful therapy for breast cancer, considerably reduce the chance of developing invasive breast cancer or its recurrence. It also can be used by people who are very susceptible to breast cancer to lower their risk of developing the illness. Nevertheless, tamoxifen is associated with several potential health risks, including blood clots, paralysis, and endometrial cancer [6]. Also, taxenes is the most used with anthracyclines as neoadjuvant chemotherapy recorded neutropathy and febrile neutropenia as adverse effects [7]. So, the search for new targeted medication with antioxidant activity is an urgent need.

Antioxidants are chemicals that neutralize and eliminate oxidative free radicals including nitric oxide, hydroxyl radical, hydrogen peroxide and superoxide anion to prevent harm to cells. It is well known that such free radicals cause damage to cells by reacting with the unsaturated fatty acids present in plasma membranes, which damages membrane proteins and reduces membrane fluidity [8]. For eliminating the effects of oxidative free radicals, antioxidants are found in natural therapies that treat Parkinson’s disease, Alzheimer’s disease, cardiovascular disease, neurological disorders, and cancers [9].

Hyalourinidases (hyases) are enzymes used to hydrolyze hyaluronic acid (HA) and classified into three different categories according to their mechanism of action. The first category of these enzymes comes from venoms and vertebrates and breaks down HA to tetrasaccharides [10]. The second category of leeches, termed as hyaluronate 3-glycanohydrolases which hydrolyze HA by yielding tetra- and hexa-saccharides. The final category comes from microorganisms and they break down HA to release unsaturated disaccharides via the β-elimination reaction [11]. It has been used for a very long time to increase the absorption of drugs into tissue and reduce tissue damage when a medication seeps into surrounding tissue [12]. Several organs in human body contains hyase enzyme such as testises, kidneys, spleen, uterus, skin, liver, eyes and placenta [13]. It was found also in body fluids, such as blood, tears, and seminal fluid and in microorganisms, such as bacteria, yeast, and fungi [14]. Bacterial hyase can be obtained from variety sources of microorganisms belonging to several genera, such as *Micrococcus*,* Streptococcus*,* Streptomyces* and *Clostridium* [15–17]. The clinical significance of hyase enzyme is well recognized in different fields including orthopaedics, ophthalmology, surgery, cardiology, internal medicine, dermatology and anticancer therapies. In cancer proliferation hyaluronic acid, the main component of the cellular matrix, attach to the CD44 marker on cancer cells triggering several biological activities. HA and CD44’s connection encourages epidermal growth factor receptor-mediated pathways, which therefore results in the growth of tumor cells, tumor cell migration, and chemotherapy resistance. Numerous investigations have demonstrated that hyaluronic acid volume in tumors is often larger in malignant than in normal tissues [18]. In addition, high concentrations of HA, a very hydrophilic substance, in the tumor stroma raise interstitial pressure which stops the uptake of chemotherapeutics which will have negative effect on cancer patient. For these reasons, hyase is incredibly effective for cancer therapy in two opposite ways [19]. First way is to increase the entry of chemotherapeutic drugs into tumors by break down extracellular matrix (HA) [20]. Second way is to inhibit tumor development and progression by disrupting the connection between CD44-HA [21]. Interestingly, hyases are now employed in some medicines to increase drug diffusion [22].

Since 1940, hyases have been isolated and characterized for the first time which showing unique characteristics in a variety of biological processes including fertilization [23], migration and differentiation of Cells, wound healing, embryonic development [24], inflammation, development and spread of cancerous cells [25]. Production of hyase is significantly affected by several physical factors including incubation temperature, pH, and fermentation period. Also, it is influenced by vitamins, co-factor, hormones, and amino acids as nutritional factors. Optimization of these factors to produce hyase is essential for increasing the enzyme’s productivity [26]. Extracellular microbial hyase can be purified from protein mixture using simple consecutive steps; ammonium sulfate precipitation, membrane technologies and chromatographic techniques [27]. The aim of this work was isolation microbial hyase enzyme, purification and characterization for antioxidant and anticancer applications.

## Materials and methods

### Sample materials and bacterial isolation

Rooster comb weighed 10 g was obtained from freshly slaughtered 50 days old rooster. The comb was slightly washed with sterilized water then submerged in sterile 0.9% saline solution then immediately sent to the laboratory. Comb was sliced using sterile surgical scalpel then was applied over the surface of modified minimal salt agar medium containing (g/L): syringe filtered hyaluronic acid (pore size 0.2 μm) as the only source of carbon:1, K_2_HPO_4_: 0.35, MgSO_4_: 0.5, NaNO_3_: 2.0, FeSO_4_.7H_2_O: 0.01, KCl: 0.5, and Agar 18–20 [28] then incubated for 48 h at 37ºC. After incubation period, bacterial colonies morphologically different were detached and streaked on the surface of solidified nutrient agar plates for pure cultures. Pure isolates were preserved on agar slant for further use [29].

### Screening for microbial hyaluronidase production

The pure isolates were screened for hyase production using HA agar medium containing yeast extract (10 g/L), K_2_HPO_4_.3H_2_O (2 g/L), MgSO_4_.7H_2_O (1 g/L), HA (1 g/L), bovine serum albumin (10 g/L), agar (20 g/L), and pH was adjusted to be 7.0. Pure isolates were inoculated on HA agar plate surface then incubated at 37 °C for 24 h. Plates were fully submerged for 10 min with a solution of 2 M acetic acid after incubation. The hyaluronidase producing isolates showed transparent zones surrounding colonies which can be distinguished from a white precipitate resulted from reaction of unbroken HA with BSA. Selection of the most hyaluronidase producing isolate was based on the largest diameter of transparent zone [30].

### Morphological and biochemical characteristics of the most potent isolate

The morphological characteristics of the selected isolate were performed based on size, shape, color, margin, elevation, consistency, and pigment of the colony. Its microscopic characters were studied using gram stain [31]. Motility test was performed using semisolid deep agar method [32].

The biochemical characterization was carried out using Arya et al.‘s methodology [33] which included gelatin hydrolysis [34], casein hydrolysis [35], methyl red and Voges-Proskauer test [36], starch hydrolysis [37], indole test [38], H_2_S production, nitrate reduction [39] and carbon utilization [40]. Bacterial isolate was also examined for their enzymatic activities such as urease [41], oxidase [42] and catalase [43].

### Molecular identification

The selected isolate was firstly subjected to DNA extraction according to Liu et al. [44]. Using a forward primer (27-Fwd 5′AGAGTTTGATCCTGGCTCAG 3′) and reverse primer (1492-Rev 5′ACGGYTACCTTGTTACGACTT 3′), bacterial 16 S rRNA was amplified by polymerase chain reaction (PCR) as described by Zhang et al. [45]. The amplified 16 S rRNA was sequenced using Macrogen (https://www.macrogen.com). The obtained 16 S rRNA gene sequence was submitted via the submission portal tool of the NCBI GenBank database (https://submit.ncbi.nlm.nih.gov/subs/genebank/). The obtained sequence was examined and matched to sequences deposited in the gene bank database (http://www.ncbi.nlm.nih.gov). The neighbor-joining phylogenetic tree was then created using the MEGA11 program.

### Optimization of hyaluronidase production by the most potent isolate

The minimal salt medium containing 1 g/L hyaluronic acid was used as a basal medium for optimization of hyaluronidase enzyme production using one factor at a time methodology. Different variables were examined including incubation temperature, pH, and fermentation period. The inoculum size was 5mL of 24 h old bacterial suspension (10^6^ CFU).

### Activity assaying of hyaluronidase enzyme

Hyaluronic acid sodium salt was used in the turbidity reduction test technique to assess the enzyme activity [46]. The enzyme’s reduction turbidity was assessed by mixing 0.05 M sodium phosphate buffer with 0.05 M NaCl at pH 7.0 with 1 mL of the crude enzyme and 1 mL of substrate hyaluronic acid (100 µg/mL) then incubation for 30 min at 37 °C. After incubation, 2.5 mL of an acidified protein solution of bovine serum albumin (1% w/v) was added and allowed to sit at 37 °C for 10 min. The decrease in turbidity was evaluated at 600 nm to assess the activity of the enzyme [47].

### Optimum incubation temperature

The selected isolate was cultured in 50 ml medium Erlenmeyer flasks and incubated at different temperatures, 25 °C, 30 °C ,37 °C and 45 °C. All flasks were shaking incubated at 150 rpm for 24 h. The cell free centrifugate was obtained at 4000 rpm centrifugation for 30 min using Beckman centrifuge (Model-Avanti™ -25 Beckman Co., U.S.A) and used for enzyme assay [48].

### Optimum pH

To examine the effect of starting pH on the production of hyaluronidase enzyme, different pH values were used, 4, 5, 6, 6.5, 7, 8 and 9 in 50 ml Erlenmeyer flasks. All flasks were incubated at 30 °C at 150 rpm for 24 h [47].

### Fermentation period

The suitable fermentation period supporting the maximum production of enzyme was investigated at different periods, 16,24, 48, 72 and 96 h at 150 rpm [48].

### Production and extraction of hyaluronidase

Two liters of culture media that had been incubated under the optimum conditions (pH, 7 and 30 °C for 48 h) were centrifuged for ten minutes at 1000 rpm. Purification was applied to the cell free supernatant [49].

### Purification of the hyaluronidase enzyme

In order to purify the hyaluronidase from crude filtrate, the enzyme filtrate was firstly precipitated using 70% saturation ammonium sulfate solution as recommended by Reda & El-Shanawany [16]. The protein precipitate was dissolved using a 50 mM phosphate buffer (pH 7) and dialyzed three times using the same buffer. The dialyzed enzyme was applied to a DEAE-cellulose column (5 × 150 mm, GE Healthcare, Sweden) at 4 °C previously equilibrated with the same phosphate buffer. The bound hyaluronidase was eluted from DEAE-cellulose column using a gradient of NaCl in phosphate buffer (pH 7). After positive fractions for hyaluronidase activity were pooled and further dialyzed against phosphate buffer (pH 7), then loaded onto a Sephacryl S-100 size exclusion column (5 × 150 mm, GE Healthcare, Sweden), which was pre-equilibrated using the same phosphate buffer. Hyaluronidase was eluted using a NaCl gradient in phosphate buffer (pH, 7) ranging from 0.2 to 1.0 M. The most highly active fractions of hyaluronidase were combined, concentrated by dialyzation against 50 mM phosphate buffer (pH 7), lyophilized, and kept at -80 ºC until further uses [50].

### Determining total protein and molecular weight

The total protein in samples was conducted by the standard Bradford method [51] for hyase content calculation and bovine serum albumin (BSA) serving as the standard. Hyase purity and homogeneity were assessed using 12% SDS-PAGE (sodium dodecyl sulfate-polyacrylamide gel electrophoresis) as described by Laemmli [52]. The gel was operated at 30 volts for 10 min then at 90 volts for 2 h. The purified hyaluronidase’s molecular weight was then ascertained using protein BL Ultra pre stained protein ladder, 6.5 to 240 KDa. to compare its electrophoretic mobility after the gel had been stained with Coomassie Brilliant Blue R250. For subsequent usage, the purified enzyme was freeze-dried and stored at -20 ºC [53].

### Physiochemical characteristics of the purified hyase

#### Effect of pH on the purified hyase

The ideal pH for the purified hyase enzyme was assessed at different pHs (4, 5.8, 6, 7, 8, 9 and 10) using several buffer systems (sodium citrate-Na_2_HPO_4_ (pH 2.0–5.0), Na_2_HPO_4_-NaH_2_PO_4_ (pH 6.0–8.0), and Na_2_CO_3_-NaHCO_3_ (pH 9.0–10.0) as reported by Guo et al. [30]. The turbidity reduction test was used to measure enzyme activity as mentioned above.

#### Effect of temperature on the purified hyase

Different temperatures of 15 °C, 20 °C, 30 °C, 37 °C, 45 °C, and 50 °C were employed to study the optimum temperature for the hyase activity [47].

#### Thermal stability of the purified hyase

Thermal stability of the purified hyase was assessed by the incubation at various temperatures (20 °C, 30 °C, 40 °C and 50 °C) for varying times (20, 30, 40, and 50 min) using a heat-controlled water bath. After incubation, samples were cooled rapidly using ice and the activity of hyase enzyme was assayed under ideal conditions [16].

### The cytotoxicity of the purified hyaluronidase on normal and cancer cell lines using MTT assay

The cytotoxicity of the purified enzyme was assessed on normal Human Skin Fibroblasts (HSF) and four cancer cell lines; Caco-2 (colon cancer), A549 (lung cancer), A431 (skin cancer), and MDA (breast cancer) using colorimetric MTT, 3-(4,5-dimethylthiazol-2-yl)-2,5 diphenyltetrazolium bromide, assay [54]. Three 96-well plates were inoculated with normal and cancer cell lines and incubated for one day. DMEM culture medium (Lonza, USA) supplemented with 10% fetal bovine serum (FBS) was used for A549 and MDA cells, while for Caco-2 and A431 cells, the medium was supplemented with 5% FBS. The cells were exposed for the purified hyase at doses of 6.25, 12.5, 25, 50, 100, and 200 µg/mL in triplicates, then incubated at 37 °C and 5% CO_2_ for 24 and 72 h. After treatment, cells were washed 3 times with fresh media and MTT solution (0.5 mg/mL in PBS, pH 7.2) was added and the cells were incubated for further 3 h. MTT solution was discard and 200 µL of dimethyl sulfoxide (DMSO) was added to each well. After measuring the absorbance at 570 nm, GraphPad Prism 7.0 software was utilized to determine the half maximum inhibitory concentration (IC_50_) of the purified hyase. The selectivity index (SI), calculated by dividing each cancer’s IC_50_ by the normal HSF IC_50_, for all cancer types were investigated [55]. Moreover, the morphological changes in the normal and cancer cell lines after treatment with the purified hyase at three doses of 12.5, 25, and 50 µg/mL was examined using phase contrast microscopy (Olympus, Germany) in comparison with the untreated control cells.

### Apoptosis changes using nuclear staining analysis

The ability of the purified hyase to cause apoptosis in the breast MDA cancer cells at different concentrations (12.5, 25, and 50 µg/mL) was investigated using fluorescence nuclear staining which utilize propidium iodide dye and ethidium bromide/acridine orange (EB/AO) (Sigma Aldrich) [56].

### Cell cycle analysis

The hyase-treated MDA cells at three concentrations (12.5, 25, and 50 µg/mL) were examined for cell cycle distribution using the flow cytometry approach [57].

### Quantitative analysis of oncogenes expression changes

The total RNA of the hyase-treated MDA cells at IC_50_ dose, 5-FU (5-fluorouracil) treated-MDA cells (as a positive control) and untreated MDA cells were extracted using Gene JET RNA purification kit (Thermo Scientific). Using mRNA, a cDNA was synthesized by using SYBR Green Real-Time PCR Master Mix and specific primers for selected genes. The used primers for Bcl-2 gene detection were 5′-ATGTTTTGCCAACTGGCCAAG-3′ (forward) and 5′-TGAGCAGCGCTCATGGTG-3′ (reverse) while for p21 gene were 5′-CCACAGCGATA TCCAGACATTC-3′ (forward) and 5′- GAAGTCAAAG TTCCACCGTTCTC − 3′(reverse). The following primers forward/reverse were used for p53 and Caspase9 genes; 5′- TCCGATCAGGAAGGCTAGAGTT-3′/5′-TCGGTCTCCTAA-AAGCAGGC- 3′ and 5’- ATTGCACAGCACGTTCACAC-3’/5’- TATCCCA TCCCAGGAAGGCA-3’, respectively. Using the formula of 2^−∆∆CT^ (2ˆ(− delta delta of the threshold cycles (CTs)), the change in the expression levels of tested genes expression were determined in the treated cells compared to untreated cancer cells [58].

### Tumor markers and inflammatory cytokines

The levels of tumor marker proteins (Caspase-9 and Caspase-3) and inflammatory cytokines (tumor necrosis factors (TNF- α) and interlukin-1 beta (IL-1β)) were determined using reagent ELISA-kit (Sino Gene Clon Biotech Co., Hang Zhou, China) and ELISA microplate reader (Dynatech Microplate Reader Model MR 5000).

### Antioxidant properties of the purified Hyaluronidase

The potential antioxidant of the purified hyase was determined using scavenging assay of DPPH free radical [59] and total antioxidant capacity [60] using different enzyme concentrations of 10, 20, 30, 40 and 50, 100 and 200 µg/mL in triplicate.

### Statistical analysis

All data were analyzed via a one-way ANOVA then a Duncan’s multiple range test at a probability level < 0.05 (*P* < 0.05) using CoStat software program Version 6.303 copyright (c) 2004.

## Result and discussion

### Microbial isolates for hyase production

**Fig. 1:**
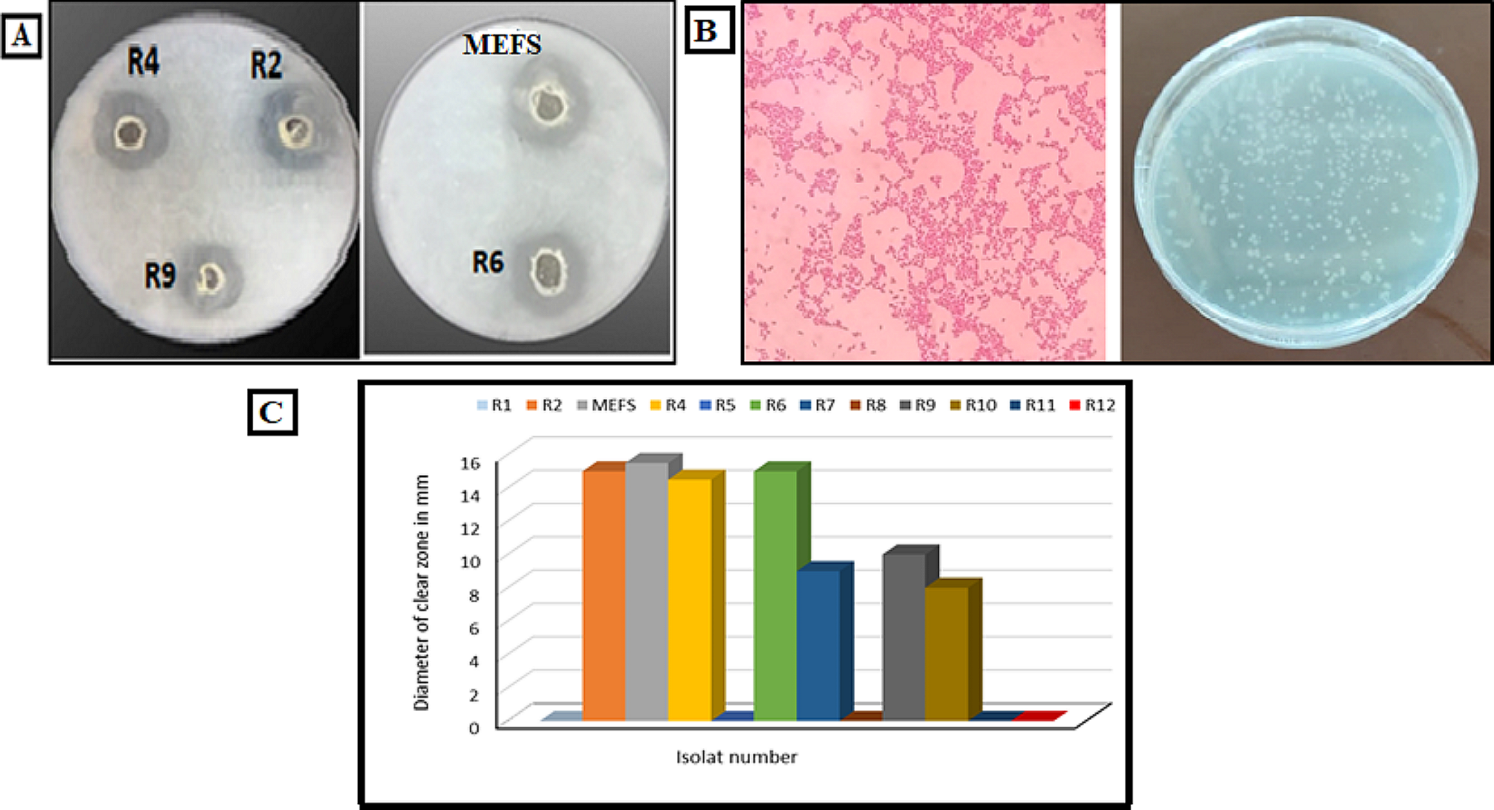
**(A)** Transparent zones for hyase production by bacterial isolates on HA agar medium. **(B)** Diameter of transparent zones of hyaluronidase producing isolates. **(C)** Morphological characteristics of MEFS isolate on nutrient agar medium and Gram stain under light microscope

Eleven bacterial isolates and one actinobacterium were isolated from rooster comb for hyase production. Seven isolates showed good hyase activity (Fig. 1C) and MEFS isolate was selected as the most potent isolate for hyase production based on the diameter of hydrolyzed zone (Fig. 1A). Hydrolysis zone method was used by many researchers as a primary screening for hyaluronidase enzyme production [30, 61, 62].

### Identification of the most potent isolate

Based on the cultural characteristics, MEFS isolate can grow well on nutrient agar medium and produce beige color with smooth texture and no external pigment. The obtained morphological features showed that MEFS is a G-ve rod bacterium not forming spores or capsulated. The biochemical characters of MEFS isolate were presented in Table 1. MEFS isolate can produce some enzymes such as nitrate reductase, oxidase, catalase, and urease. Moreover, MEFS isolate can utilize glucose, maltose, fructose, mannose and arabinose as carbon sources.

**Table 1:** Morphological and biochemical characteristics of MEFS isolate

Characteristics	Response
**(1) Cultural characteristics**:	
Growth Color Diffusible pigment Texture	Good beige -ve smooth
**(2) Morphological characteristics**:	
Gram stain Spore stain Capsule stain	Gram -ve rod -ve -ve
**(3) Biochemical characteristics**:	
Nitrate reduction Starch hydrolysis Gelatin liquefaction H_2_S Catalase test Blood hemolysis Oxidase Methyle red VP (Voges-Proskauer) Indole test **Hydrolysis of** Casein Urea	+ - - - + - + - - - - +
**Utilization of different carbon sources**	
No Carbon Glucose D-galactose maltose Fructose Mannose D-xylose L-arabinose Erythritol	- + - + + + - + -

### Molecular identification

**Fig. 2:**
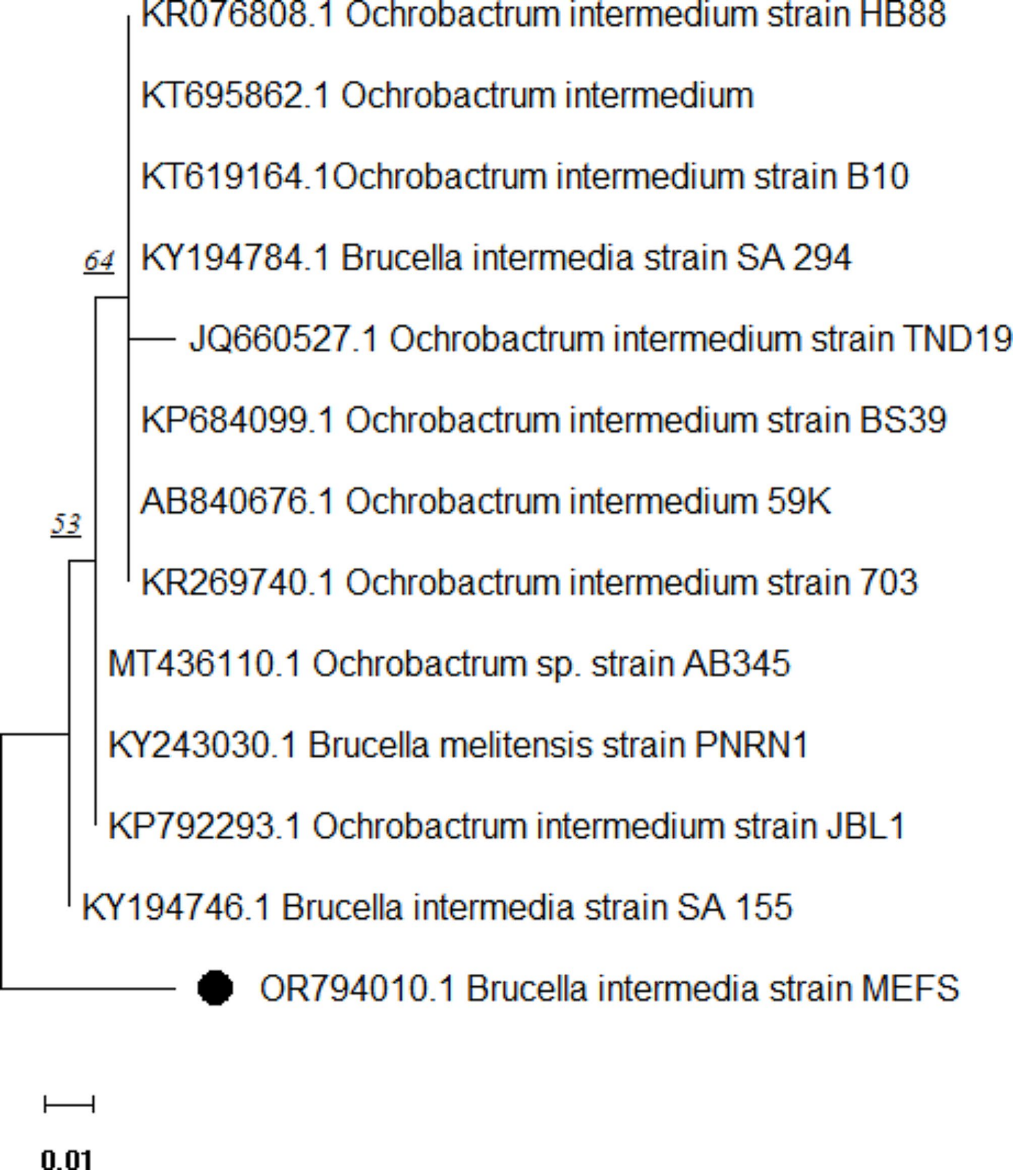
The phylogenetic tree of *Brucella intermedia* MEFS strain using MEGA11 software

16 S rRNA gene sequences of MEFS isolate was submitted to GenBank with coded number OR 794,010. Based on the known 16 S rRNA sequences in NCBI, homologous analysis showed that the strain MEFS had 99% similarity with *Brucella intermedia*. Based on the strain MEFS’s 16 S rRNA gene sequences with its neighbors, a phylogenetic tree was built to reveal that the MEFS strain formed a distinct phyletic line close to *Brucella intermedia* (Fig. 2).

## Optimization of hyaluronidase production

### Effect of incubation temperatures

**Fig. 3:**
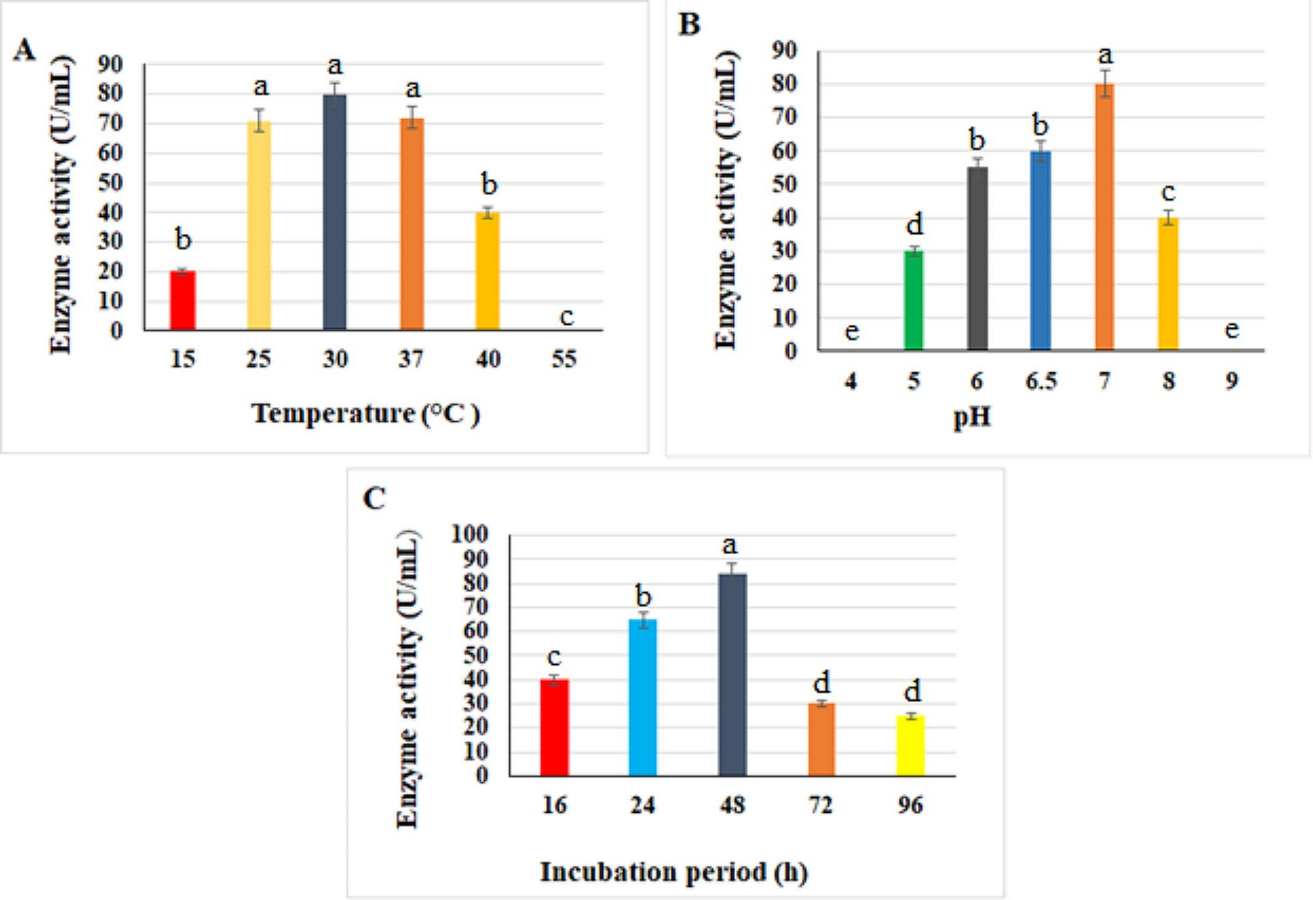
Optimization of hyaluronidase enzyme production by *Brucella intermedia* MEFS. **(A)** Temperature, **(B)** pH and **(C)** incubation period. Significant differences are indicated by different letters between the bars at *p* < 0.05 using Duncan’s multiple range test

The optimum temperature was investigated by incubating *Brucella intermedia* MEFS at different temperatures and the maximum production of hyase enzyme (80 U/mL) was obtained at 30 °C (Fig. 3A). Further elevation in incubation temperature resulted in low production of enzyme due to its denaturation [27]. Patil et al. [47] recorded that the maximum production of hyase enzyme (284 U/mL) was at 37 °C. Mahesh et al. [48] also reported that *Streptococcus mitis* recorded the highest production of enzyme at mesophilic range of temperature.

### Effect of initial pH

Based on data present in Fig. (3B), the optimum pH for hyaluronidase was at 7 with enzyme activity of 80 U/mL. However, at pH 4 and 9 there was no production of hyaluronidase enzyme. Medium pH is essential factor in the production of any metabolic substrate as it influences the properties of the medium, solubility of materials and ionic state of hyaluronic acid as a bacterial substrate [61]. Neutral pH was recorded for hyaluronidase optimum production by Patil et al. [47] and Kadhum [63], who in consent with our results. In contrast, Sahoo et al. [61] recorded the optimum production of hyaluronidase at pH 5.5.

#### Effect of various incubation periods

The time course for hyaluronidase production showed the maximum activity of enzyme (84 U/mL) was within 48 h (Fig. 3C). The same results were obtained by Sahoo et al. [61] who found that the highest production of hyase by *Streptococcus mitis* was after 48 h incubation.

#### Purification of the hyase

**Fig. 4:**
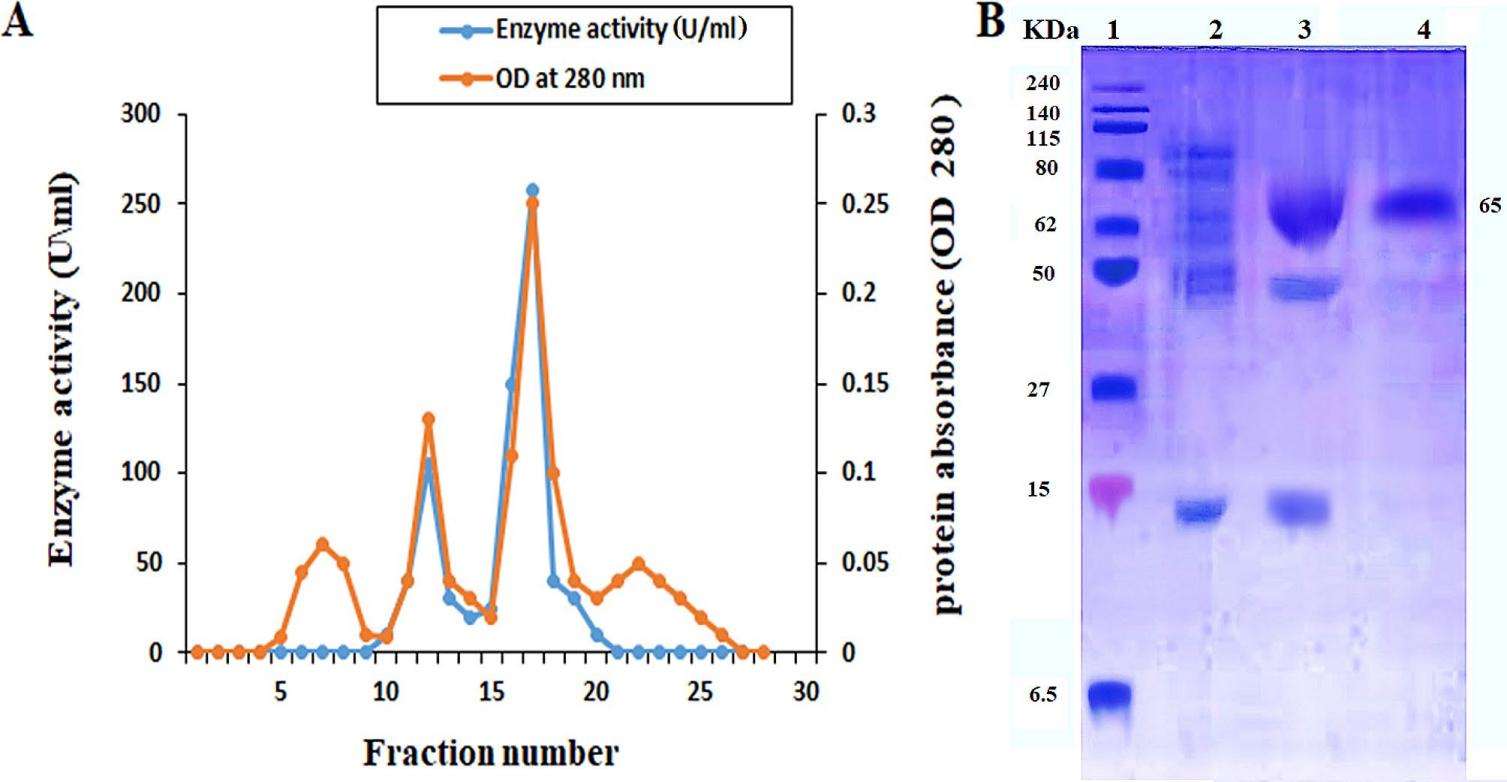
**(A)** Hyaluronidase elution profile on a DEAE-cellulose column that has been previously equilibrated with phosphate buffer (50 mM at pH 7). The red line on the left vertical axis represents the protein (Absorbance at 280 nm), while the blue line on the right vertical axis represents the hyaluronidase activity (Unit / ml). **(B)** 12% SDS-PAGE electrophoresis during hyaluronidase purification steps: A protein marker was shown in lane 1, crude enzymes were shown in lane 2, bands eluted from a DEAE column were shown in lane 3, and Lane 4 displayed the purified hyase, which has 65 KDa as a relative molecular weight

The initial specific activity of hyase enzyme in the crude filtrate of the *Brucella intermedia* MEFS strain was estimated to be 2 U/mg protein (Table 2). The specific activity rose to 3.05 U/mg protein after salting out and estimated to be 10.32 and 18.58 U/mg protein with 806% and 69.7% yield and 5.2 and 9.3-fold increase over the initial crude enzyme after ion-exchange and gel filtration chromatography, respectively (Table 2). In ion exchange chromatography, hyaluronidase eluted from previously equilibrated DEAE cellulose column with NaCl at the gradient of 0.6 and 0.8 M as its elution profile. Figure (4 A) showed two maximum hyase activity peaks ranging at 105 and 258 U/mL for fractions numbers 12 and 17. Purification of hyase enzyme using DEAE cellulose and Sephacryl columns was recommended by Abdel-Monsef et al. [64] as a simpler and faster method. Using salting out with ammonium sulfate, ion-exchange, and gel filtration chromatography, Reda & El-Shanawany [16] isolated the hyase enzyme from *Streptomyces roseofulvus* with specific activity of 3.2 U/mg protein, 31.4% final yield, and 9.2-fold. Another report on the purification of hyase from the *Bacillus sp*. A50 strain found that the enzyme has 102.14 U/mg protein, 25.38%, and 21-fold for specific activity, yield and fold, respectively [30].

**Table 2:** The purification profile of hyaluronidase enzyme produced by *Brucella Intermedia*

Purification step	Total Protein (mg)	Total activity (U/mL)	Specific activity (U/mg)	Yield	Purification fold (%)
Crude filtrate	160	320	2	100	1
70% Amm. sulfate Precipitation	95	290	3.05	90.6	1.53
DEAE–cellulose column	25	258	10.32	80.6	5.2
Sephacryl S-100 (Pure hyaluronidase)	12	223	18.58	69.7	9.3

## Molecular weight of the purified hyase

The purified hyase enzyme revealed a single molecular weight protein band of roughly 65 KDa on 12% SDS-PAGE gel electrophoresis (Fig. 4B). Among bacterial hyases, a wide range in its molecular weight was reported. Hynes & Walton [14] revealed that the molecular weights of hyase purified from *S.pneumonia*, *S.agalactiae*, *Clostridium perfringens*, *Streptococcus aureus*, *Propionibacterium acnes* and two enzymes *from Streptomyces sp* were107, 121, 114 ,92, 82& (77,84) KDa, respectively.

## Characterization of purified Hyase

### pH impact on hyase activity

**Fig. 5:**
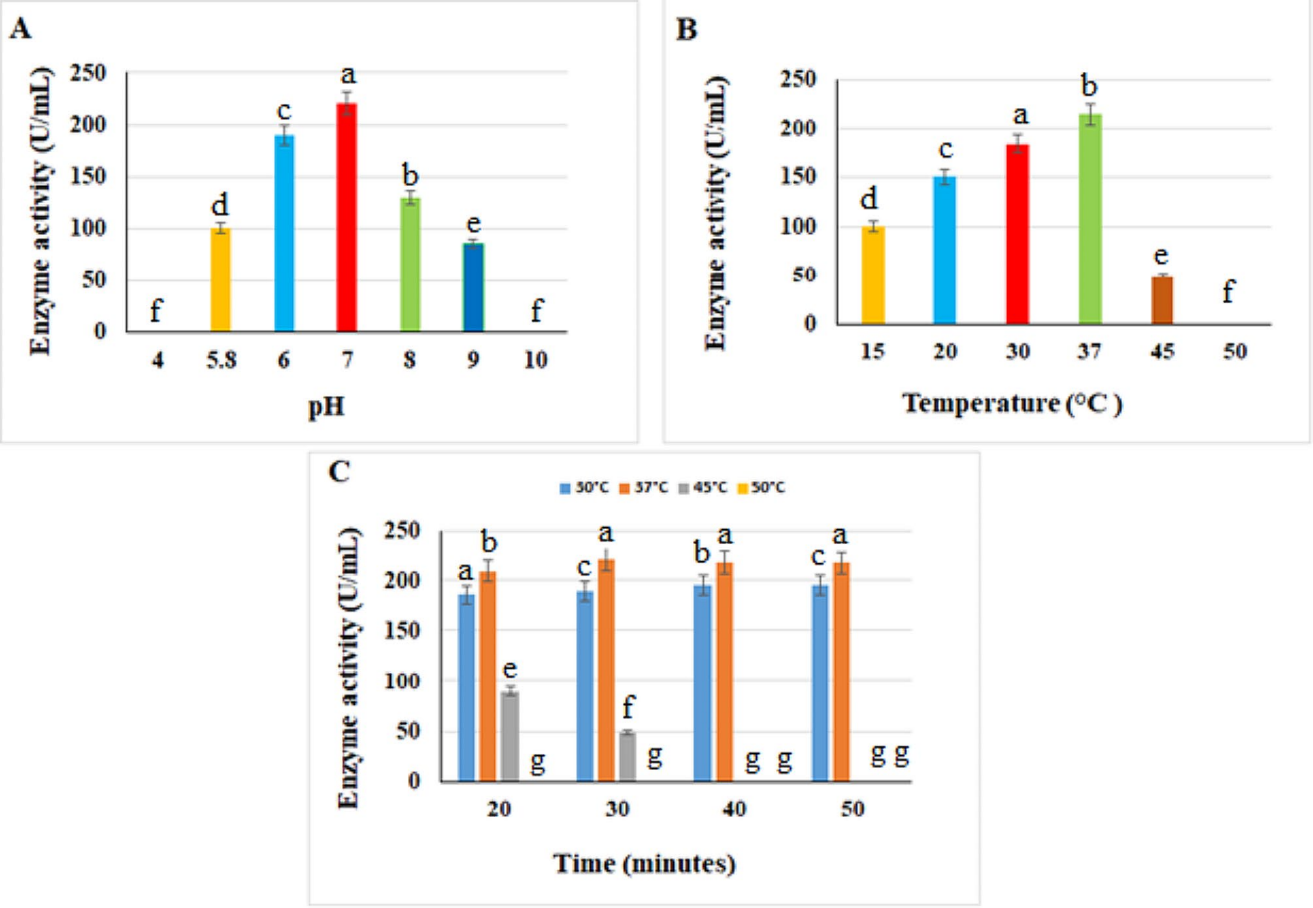
Effect of different pHs and temperatures on hyase activity. **(A)** Effect of pH, **(B)** Effect of temperature and **(C)** Hyase thermal stability at different times. Significant differences are indicated by different letters between the bars at *p* < 0.05 using Duncan’s multiple range test

The data presented in Fig. (5 A) revealed that hyase activity declined at pH values of 5 and 9, and it completely disappeared at pH 4 and 10. Hyase’s maximal activity was at pH 7 (220 U/mL), indicating that the purified hyase enzyme displayed a wide range of pH. According to Guo et al. [30], the ideal pH for hyase activity purified from *Bacillus* sp A50 strain was 6.5. Reda & El-Shanawany [16] reported the maximum activity of *S. roseofluvus* S10 hyase (3.91 U/mL) was at pH 9.

#### Temperature impact on hyase activity

The activity of the purified hyase was tested for optimum temperature at pH 7. The results in Fig. (5B) showed that the purified hyase degrades HA at its maximum rate of 215 U/mL at 37 °C, whereas activity sharply decreases at 40 °C. The enzyme became denaturized at temperatures as high as 50 °C, at which it lost all its activity. These results were consistent with Reda & El-Shanawany [16] who recorded the temperature gave the highest hyase activity (4.99 U/mL) at 35 °C.

### Thermal stability of the purified hyase

The purified hyase enzyme exhibited stability and activity within the temperature ranged from 30 °C to 45 °C for 30 min (Fig. 5c). Its activity was stable at 30–37ºC for 50 min and the enzyme totally lost its function at 50 °C. These findings were consistent with that of Patil et al. [47] who revealed the purified hyase enzyme maintained its stability and 100% activity within the 10–40 °C and a substantial decline in its activity was recorded above 40 °C. The purified hyase enzyme demonstrated stability and high activity at 37 °C. This finding suggests that this enzyme may have significant applications in medicine.

#### Applications of hyaluronidase enzyme

In vitro**anticancer activity of the purified hyase**.

**Fig. 6:**
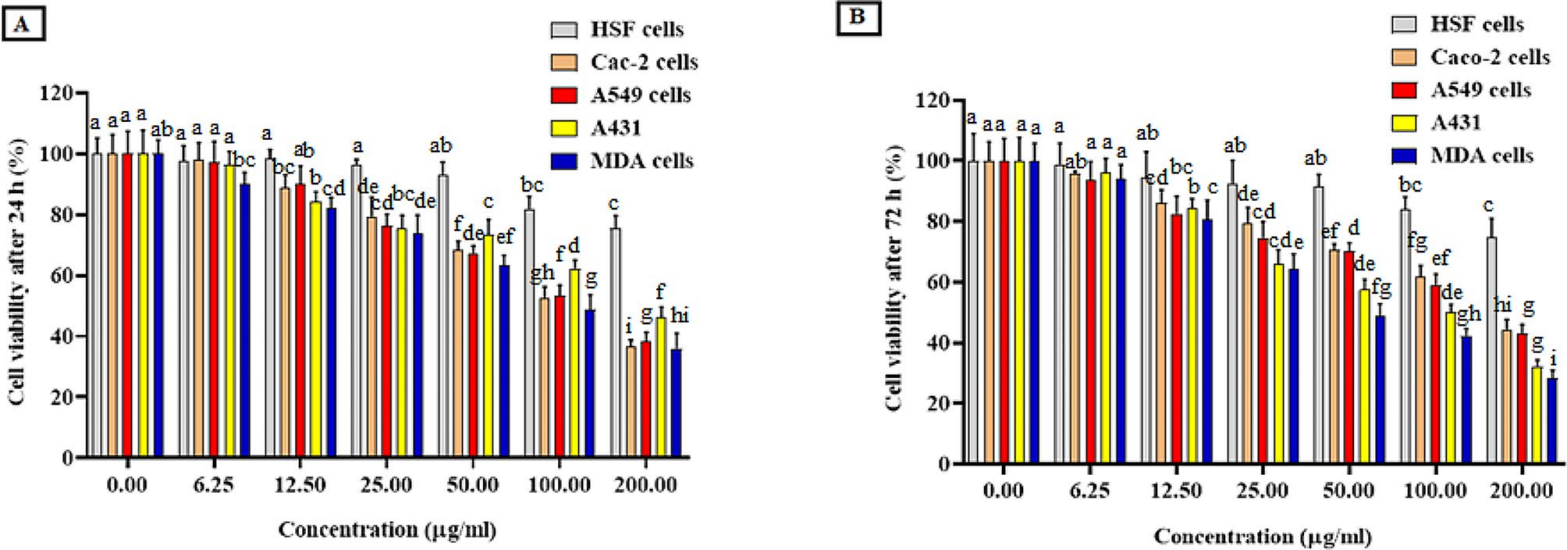
Cytotoxicity of the purified hyase on cancer and normal cell lines using MTT assay. **(A)** Cell viability after 24 h. **(B)** Cell viability after 24h72 h. The mean values from three experiments expressed as mean ± SE. Significant differences are indicated by different letters between the bars at *p* < 0.05 using Duncan’s multiple range test

The cytotoxicity of hyase enzyme against normal HSF cells and tumor A431, Caco-2, A549, and MDA cell lines was evaluated. The results in Table (3) and Fig. (6) indicated an improvement in the biosafety of the hyase on HSF cells after 24 h and 72 h, where the maximum IC_50_ values were 310.9 ± 19.59 and 276.1 ± 16.36 mM, respectively. Similarly, hyase significantly improved its selectivity and toxicity on Caco-2, A431, A549 and MDA cells, with maximum IC_50_ values of 89.34 ± 4.68 and 72.88 ± 4.04 mM for Caco-2 cells after 24 h and 72 h, respectively, and the lowest IC_50_ value of 55.66 ± 5.42 mM and 31.52 ± 5.95 for MDA cells after 24 h and 72 h. Anticancer activity and inevitable damage to normal cells are factors that make the therapeutic index an important metric. Selectivity index is the first step to ensure the medication’s safety. The ratio represents the concentration of hyase at which 50% of cytotoxicity in the normal cell line occurs and the concentration of the enzyme at which 50% of cancer cell death occurs in the cancer cell line [5]. The selectivity index of hyase against MDA cell line is acceptable which indicates the biosafety and potential activity of hyase enzyme as anticancer agent.

**Table 3:** Effect of hyaluronidase enzyme (µg/ml) on cell viability percentage of IC_50_ value (µg/mL) and selectivity index (SI) on normal human skin fibroblast cells (HSF), colon cancer cells (Caco-2), lung cancer cells (A549), skin cancer cells (A549) and breast cancer cells (MDA) after treatment for 24 and 72 h

Cell line	Hyase treatment			
	24 h		72 h	
	IC_50_ (µg/mL)	SI	IC_50_ (µg/mL)	SI
HSF	310.9 ± 19.59	-	276.1 ± 16.36	-
Caco-2	89.34 ± 4.68	3.48 ± 0.21	72.88 ± 4.04	3.79 ± 0.22
A549	73.02 ± 5.21	4.26 ± 0.27	54.12 ± 5.11	5.10 ± 0.3
A431	61.23 ± 4.46	5.08 ± 0.32	37.40 ± 3.53	7.38 ± 0.44
MDA	55.66 ± 5.42	5.59 ± 0.35	31.52 ± 5.95	8.76 ± 0.52

All values were mean ± SE.

**Fig. 7:**
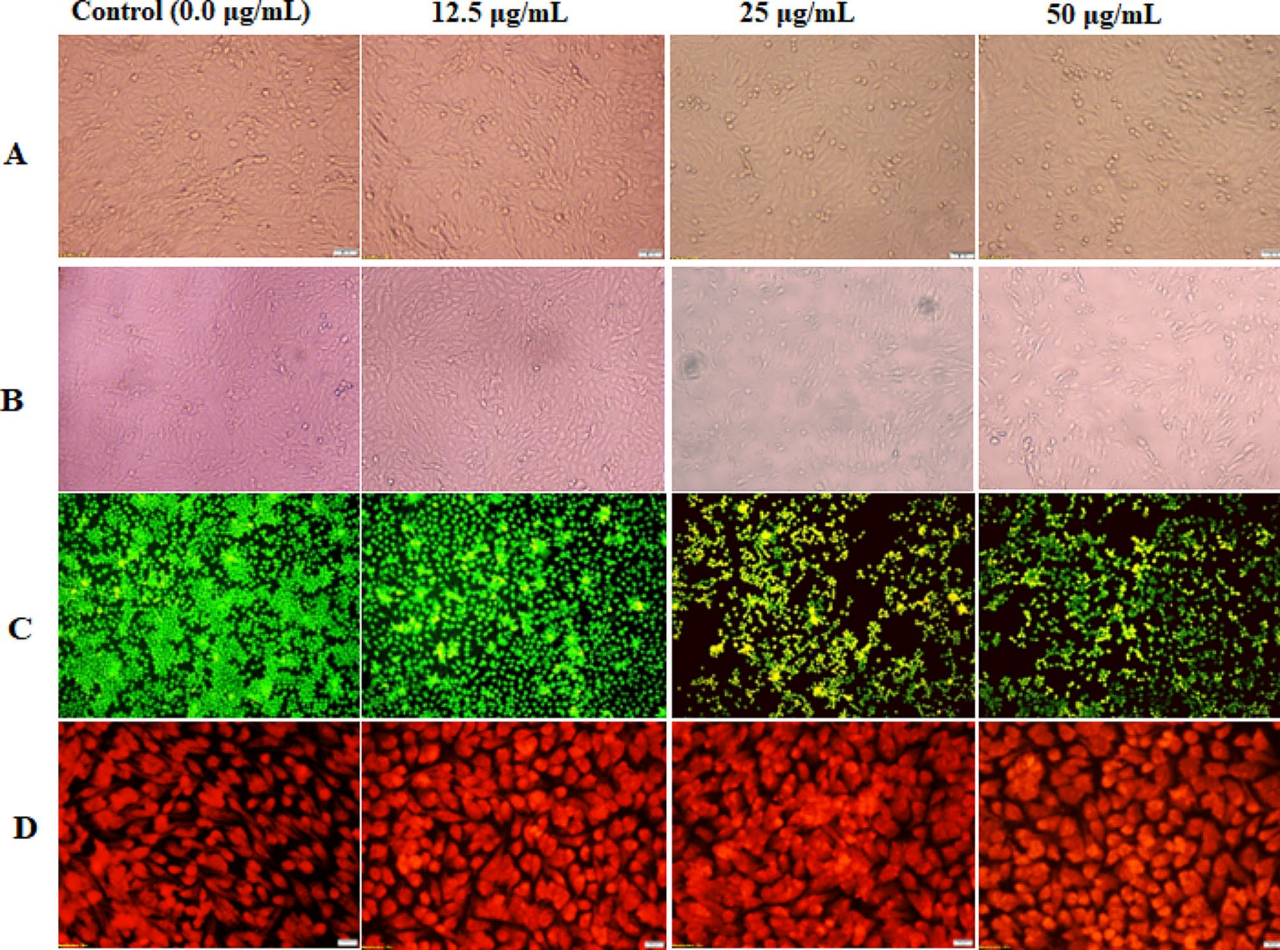
Morphological changes of hyaluronidase-treated MDA cells for 72 h at concentrations of 12.5, 25 and 50 µg/mL as compared to untreated cells and normal cells. **(A and B)** Changes in the morphology of the hyase-treated normal skin HSF cells and breast cancer MDA under phase contrast microscope, respectively. **(B)** The treated MDA cells were imaged using ethidium bromide/acridine orange fluorescence microscopy. The images showed reddish/orange fluorescence, which, in turn, indicated late apoptotic nuclei. **(C)** Reddish orange fluorescence indicates late apoptotic nuclei under propidium iodide fluorescence microscope. Magnification was 20 X and 20 μm scale bar)

Following treatment with different doses of the purified hyase, the unchanged cell morphology of normal human skin fibroblast (HSF) and morphological changes in the treated breast cancer (MDA) cells were demonstrated in Fig. 7A and B, respectively. Treatment with the purified hyase resulted in considerable nuclear condensation and tumor cell shrinkage. Furthermore, a fluorescence phase contrast microscope was used to demonstrate the vitality of MDA cells, as shown in Fig. 7C and D. Compared to untreated cells, the images showed a decrease in cell viability after hyase treatment, with complete destruction of living cells. This demonstrated hyase’s effectiveness against MDA cells as an anticancer agent, which may be related to its ability to induce apoptosis (demonstrated by an increase in orange-red cells). Acridine Orange/Ethidium Bromide (AO/EB) double staining of MDA cancer cells and (reddish orange indicate late apoptotic nuclei) upon staining with propidium iodide (Magnification 20 X; scale bar 20 μm**).** These findings were consistent with those of Thirumurthy et al. [5] who reported that in enzyme-treated cells at a concentration of 40 µg/ml, the morphology of apoptosis in cells undergoing pyknosis, chromosomal condensation, and nuclear fragmentation were visible under a microscope.

#### Cell cycle analysis

**Fig. 8:**
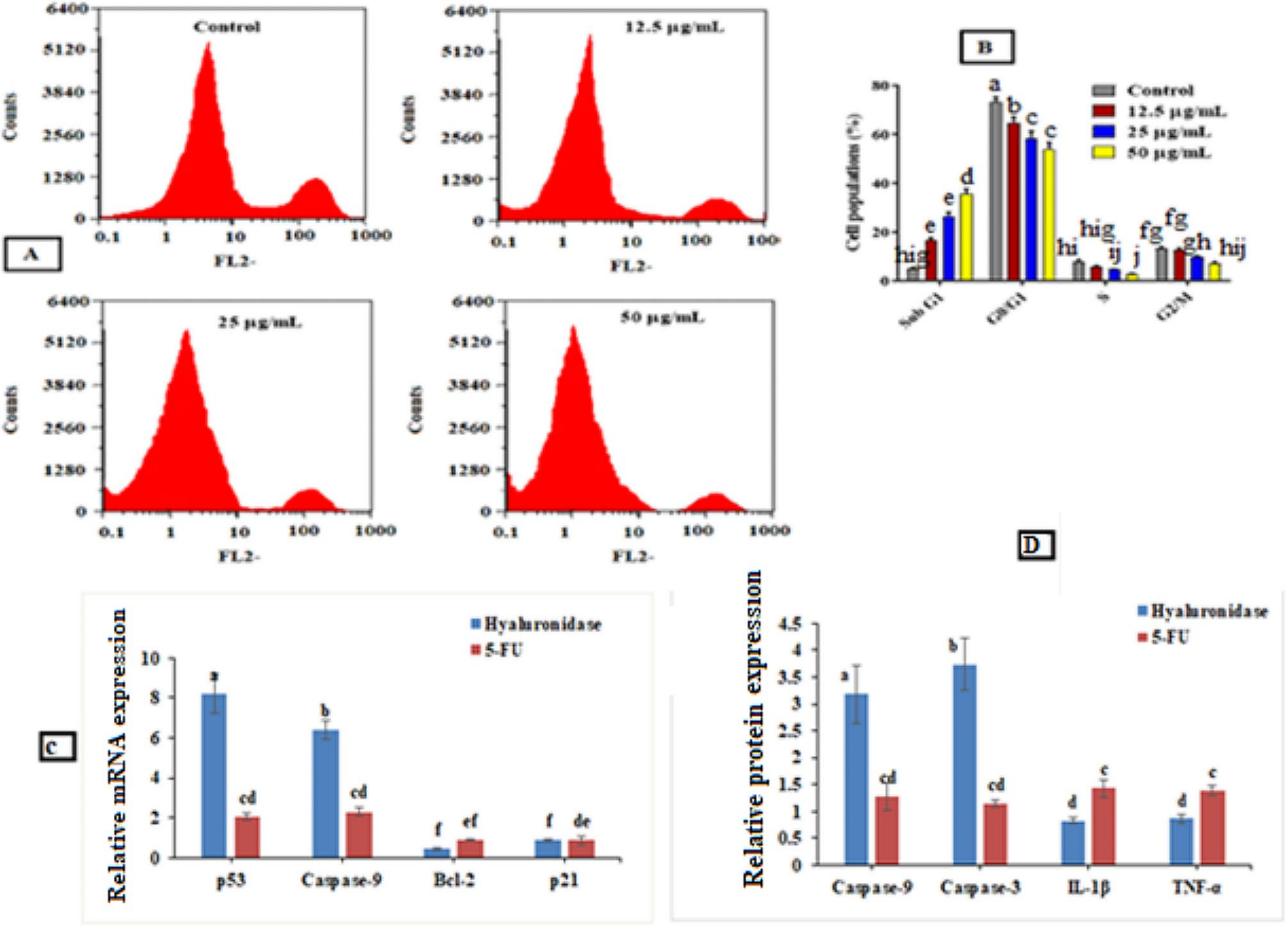
**(A)** Flow cytometry charts of cell cycle diagrams of hyaluronidase treated -MDA cells at different concentrations and control for 72 h. **(B)** Cell population of hyaluronidase treated cells in comparison to untreated cells during cell cycle stages. **(C)** Changes in p53, Caspase-9, Bcl2, p21, caspase 3, IL-1β, and TNF-α genes expression of hyase-treated cells for 72 h compared with 5-fluorouracil (5-FU) measured by real-time quantitative PCR. **(D)** Changes in caspase-9, caspase 3, IL-1β, and TNF-α protein expression of hyase-treated cells for 72 h compared with 5-FU measured by real-time quantitative PCR. The values are given as mean ± SE. Significant differences are indicated by different letters between the bars at *p* < 0.05 using Duncan’s multiple range test

Before cell division, nuclear DNA is duplicated as part of the closely controlled process known as cell cycle progression. Therapeutic substances can effectively target the regulatory systems that govern this process, which are frequently upset in tumor cells. Most cells are normally quiescent and do not divide unless they receive a signal to do so to enter the cell cycle’s active phases. Several disease conditions such as cancer, psoriasis, and hyperplasia cause a reduction in or disruption of this control [65]. To determine whether hyase regulates cell cycle progression, we used flow cytometry to examine cell cycle stages. Comparing the treated MDA cell cycle to the untreated cells, the results revealed a notable change in the cell population percentage of cancer cells upon treatment with hyase enzyme (Fig. 8A, B). Treatment with hyase notably decreased the cell populations in G0/G1 phase (the phase in which the cell is getting ready to divide) with obvious increases in the subG1 phase populations relative to untreated cells. Furthermore, as compared to untreated cells, the cell population percentages in the S and G2/M stages showed no discernible variations. This means that the purified hyase affects cancer cell population during growth phase (G1). For this reason, nearly no population appeared in S phase, which indicates the efficiency of the purified hyase as anticancer drug. These results were like that of Tan et al. [66] who recorded a reduction in populations of G0/G1 phase and an increase in populations of S phase with no alterations in G2/M.

#### Quantitative analysis of oncogenes expression changes

Carcinogenesis includes a series of steps associated with genetic changes that impact important cellular processes related to growth and development. Oncogenes are genes whose activation leads to cancer development. Drugs targeting these oncogenes gained much attention in recent years and now in clinical use. Oncogenic elements of the cell signaling system, including the cascade, have received a lot of attention in relation to breast cancer [67]. At the molecular level, hyase-treated MDA cells resulted in a considerable down-regulation of the expression of some proteins and genes, such as IL-1β, TNF-α, p21, and Bcl-2 (Fig. 8 C and D). It was found that the expression of TNF-α and IL-1β stimulate tumor cell proliferation, invasion, metastasis, and immune system subversion, which contribute to inflammation-associated carcinogenesis [68]. Moreover, Lippitz et al. revealed a correlation between elevated levels of IL-1β and a poor prognosis for cancer patients [69]. Remarkably, the expression of the genes and proteins of p53, caspase-9, and caspase-3 as tumor suppressor genes significantly increased in hyaluronidase-treated cells (Fig. 8 C and D). Researchers recorded the crucial role of p53 gene in both apoptosis and growth arrest during stressful conditions [70]. Therefore, the upregulation of p53 in conjunction with downregulation of Bcl-2 demonstrated hyaluronidase’s noteworthy capacity to cause apoptosis in the MDA cell line. According to these results, it was found that hyaluronidase helps in overexpression of tumor suppressor genes, at the same time decreasing the expression of tumor activator genes (oncogenes) as compared with 5-FU (effective anticancer drug) which prevent cancer cell growth and metastasis. This result is consistent with prior studies which revealed that apoptosis induced by different anticancer agents is related to expression of some tumor suppressor genes such as p53, Caspase 3 and Caspase 9 and reduction of some genes (tumor activator genes) such as Bcl-2 and P21 [56, 71, 72]. Based on results of cytotoxicity assay, therapeutic index cell cycle, and oncogenes recommend use of hyase enzyme as a targeted medication in cancer therapy. Some limitations of this study were the lack of previous work on the effect of hyaluronidase on the inflammation-associated carcinogenesis genes and apoptosis induction genes, and cell cycle analysis. The present study created a new strategy and giving a promising scenario for combating different types of tumors, especially breast tumor. However, further studies on the properties of *Brucella intermedia* hyaluronidase and understanding the exact mechanisms of action and in-vivo anticancer studies are needed.

#### In vitro antioxidant properties

**Fig. 9:**
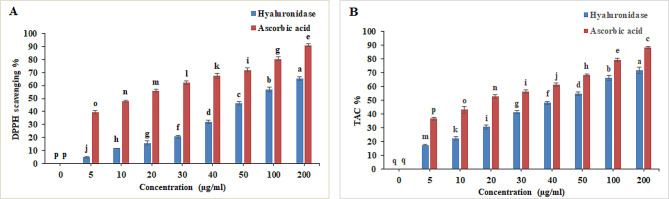
Antioxidant behavior of hyaluronidase enzyme at different concentrations. **(A)** DPPH scavenging, **(B)** Total Antioxidant Capacity % (TAC%). Significant differences are indicated by different letters between the bars at *p* < 0.05 using Duncan’s multiple range test

The data presented in Fig. (9) showed that *Brucella intermedia*’s hyase enhanced the antioxidant capabilities of DPPH radical scavenging with IC_50_ value equal 67.13 U/mL compared with the reference compound ascorbic acid of 12.45 µg/ml. For the total antioxidant capacity, the purified hyase enzyme recorded IC_50_ value of 50.59 U/mL, where ascorbic acid recorded 17.05 µg/mL. The antioxidant activity of the purified hyase was reported in previous works [73, 74]. Increasing concentration of the purified hyase led to an increase in its overall antioxidant activity. Total antioxidant activity of the purified hyase was determined to be 18, 22, 31, 42, 48, 54, 66, and 72% at various concentrations of 5, 10, 20, 30, 40, 50, 100, and 200 U/mL. DPPH scavenging activity of the purified hyase was estimated to be 5, 12, 16, 21, 32, 46, 57, and 66% at 5, 10, 20, 30, 40, 50, 100, and 200 U/mL, respectively. Hyaluronidase’s ability to effectively prevent the production of free radicals may also be reflected in its capacity to scavenge DPPH. This result in agree with Thirumurthy et al. [5] who assayed the hyaluronidase antioxidant activity at different concentrations by different five method and revealed that hyase is an effective antioxidant agent.

## Conclusion

*Brucella intermedia MEFS* isolated from rooster’s comb was the most potent bacteria to produce hyaluronidase enzyme. One factor at a time optimization was used for maximum production of hyaluronidase. The highest production of enzyme was supported by pH 7 and temperature 30 °C, and 48 h incubation period. The enzyme was purified as a single protein band. Its molecular weight was determined to be about 65 KDa. Temperature, pH, and thermal stability were found to have an impact on the activity of the hyaluronidase enzyme. It showed notable stability throughout a broad pH and temperature range, making it useful for several applications. The hyaluronidase showed a potential anticancer activity on four tumor cell lines with IC_50_ values ranged from 55 to89 µg/ml with low toxicity on normal fibroblast somatic cells. It resulted in cell shrinkage, nuclear condensation, and reduction in cell viability in hyaluronidase breast cancer treated cells. Also, it can down-regulate inflammation-associated carcinogenesis genes and up-regulate apoptosis induction genes in breast cancer cells. In conclusion, *Brucella intermedia* MEFS hyaluronidase offers an alternative source for use in cancer therapy as a treatment of different types of cancer with little side effects on patient compared to other chemotherapeutic agents.

## Data Availability

No datasets were generated or analysed during the current study.
